# The genome sequence of the Grey Pine Carpet, *Thera obeliscata *(Hübner, 1787)

**DOI:** 10.12688/wellcomeopenres.20005.1

**Published:** 2023-09-19

**Authors:** David C. Lees, Natural History Museum Genome Acquisition Lab, Darwin Tree of Life Barcoding collective, Wellcome Sanger Institute Tree of Life programme, Wellcome Sanger Institute Scientific Operations: DNA Pipelines collective, Tree of Life Core Informatics collective, Darwin Tree of Life Consortium

**Affiliations:** 1 Natural History Museum, London, England, UK

**Keywords:** Thera obeliscata, Grey Pine Carpet, genome sequence, chromosomal, Lepidoptera

## Abstract

We present a genome assembly from an individual male *Thera obeliscata* (the Grey Pine Carpet; Arthropoda; Insecta; Lepidoptera; Geometridae). The genome sequence is 404.7 megabases in span. Most of the assembly is scaffolded into 18 chromosomal pseudomolecules, including the Z sex chromosome. The mitochondrial genome has also been assembled and is 17.5 kilobases in length.

## Species taxonomy

Eukaryota; Metazoa; Eumetazoa; Bilateria; Protostomia; Ecdysozoa; Panarthropoda; Arthropoda; Mandibulata; Pancrustacea; Hexapoda; Insecta; Dicondylia; Pterygota; Neoptera; Endopterygota; Amphiesmenoptera; Lepidoptera; Glossata; Neolepidoptera; Heteroneura; Ditrysia; Obtectomera; Geometroidea; Geometridae; Larentiinae; *Thera*; *Thera obeliscata* (Hübner, 1787) (NCBI:txid934896).

## Background

The Grey Pine Carpet, *Thera obeliscata* (Hübner, 1787) is a moderate sized geometrid moth with a wingspan of about 28–36 mm. Its forewings are light to greyish brown, but variable: moths found toward the north and west of Britain, are often more reddish, with basal darker and median fasciae. The median fasciae varies in shape, but is always narrowed towards the trailing edge, and with its inner margin only slightly angled inward, unlike in the otherwise similar Pine Carpet *T. firmata* Hübner, 1822. It can be hard to distinguish from other *Thera* species. Morphologically, the diagnostic external difference is the male antenna, which is simple in *T. obeliscata*, and slightly serrate in *T. britannica* (Turner, 1925) ( Boyes *et al.*, 2023; Lewis, 2013).

The species is bivoltine in Britain, with flight periods between late April and mid-July, as well as between late August and early November ( Randle *et al.*, 2019). It overwinters as a larva and pupates underground ( Waring *et al.*, 2017). The Grey Pine Carpet occurs in coniferous forest as well as parks and gardens ( Waring *et al.*, 2017). The larva feeds on many conifers such as Scots Pine, Norway Spruce, Western Hemlock-spruce, Western Red-cedar, Lawson’s Cypress, Monterey Cypress and Douglas fir ( Waring *et al.*, 2017). *T. obeliscata* is a generally common or locally abundant species that is widespread in the British Isles and Channel Isles. It is widespread in the western Palaearctic including Scandinavia, but avoiding the south of Spain, with scattered records in non-oriental Asia ( GBIF Secretariat, 2023).

There are conflicting reports of population changes of Grey Pine Carpet in the UK. Despite the increase in conifer planting since the 19th century ( Waring *et al.*, 2017), the species is reported to have decreased in abundance by a substantial 47% between 1970 and 2016, and has decreased by 9% in distribution range. This is in sharp contrast to the recently colonising Spruce Carpet *T. cupressata* (Geyer, 1831) ( Randle *et al.*, 2019); whereas between 1968 and 2006, Rothamsted trap numbers of *T. obeliscata* increased on average annually by of about 0.5% ( Conrad *et al.*, 2006).

In Britain, *T. obeliscata* exhibits a single mitochondrial cluster on BOLD, BOLD:AAA7522 (25/02/2023), with up to about 1.28% intraspecific divergence reported by BOLD in Europe. This Barcode Index Number is shared with *T. britannica* (Turner, 1925), a species which nevertheless comprises a separate haplogroup on BOLD including UK examples still (mis-)identified as *T. variata* (Denis & Schiffermüller, 1775), and also encompasses hybrids with *T. variata*. BOLD:AAA7521 is a related cluster found in mainland Europe but not yet the UK, but currently it has also mixed identities comprising *T. obeliscata*, *T. cembrae* and *T. variata* (as of 25/08/2023). The genome sequence will be useful in comparison with that of its potential sister species of *T. obeliscata*, *T. britannica*, whose genome is available ( Boyes *et al.*, 2023), considering that their mitogenomes are only 1.37% pairwise divergent by measure of 658 bp of COI-5P (OX387930 vs OW618053 respectively). It will also be of use in future molecular-aided taxonomic work, in studying apparently recently separated sibling species, and also in studies of hybridisation ( Boyes *et al.*, 2023). *Thera* Stephens, 1831 is a member of the larentiine tribe Cidariini (in which Õunap *et al.* (2016) place *Thera* as sister to *Pennithera* Viidalepp, 1980 (see also Choi, 2000).

## Genome sequence report

**Figure 1.**
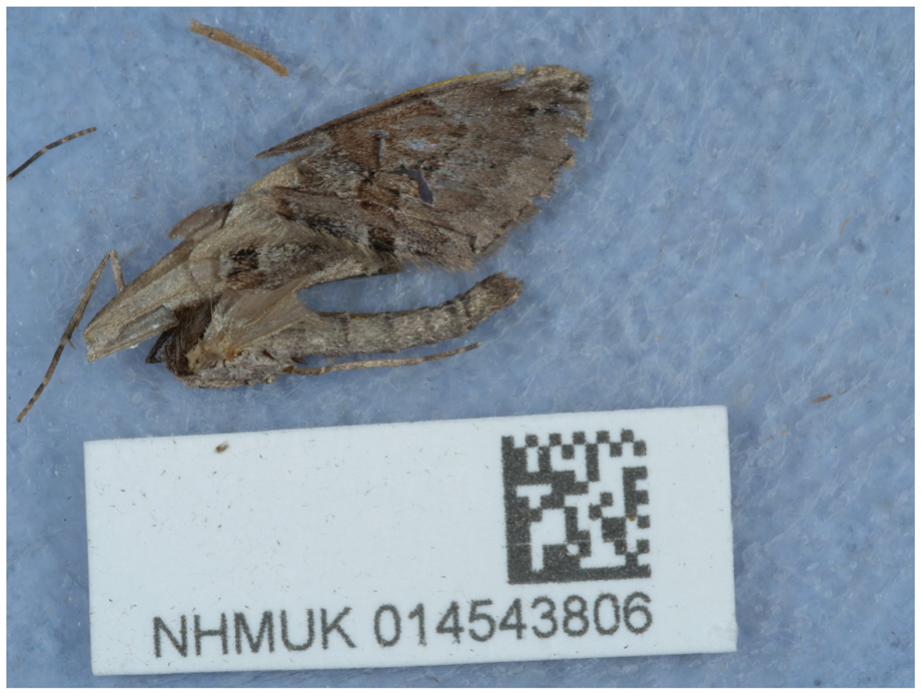
Photograph of the *Thera obeliscata* (ilTheObel3) specimen used for genome sequencing.

The genome was sequenced from one male *Thera obeliscata* ( Figure 1) collected from Beinn Eighe National Nature Reserve, Scotland, UK (57.63, –5.35). A total of 57-fold coverage in Pacific Biosciences single-molecule HiFi long reads was generated. Primary assembly contigs were scaffolded with chromosome conformation Hi-C data. Manual assembly curation corrected 19 missing joins or mis-joins and removed 2 haplotypic duplications, reducing the assembly length by 0.65% and the scaffold number by 11.54%.

**Figure 2.**
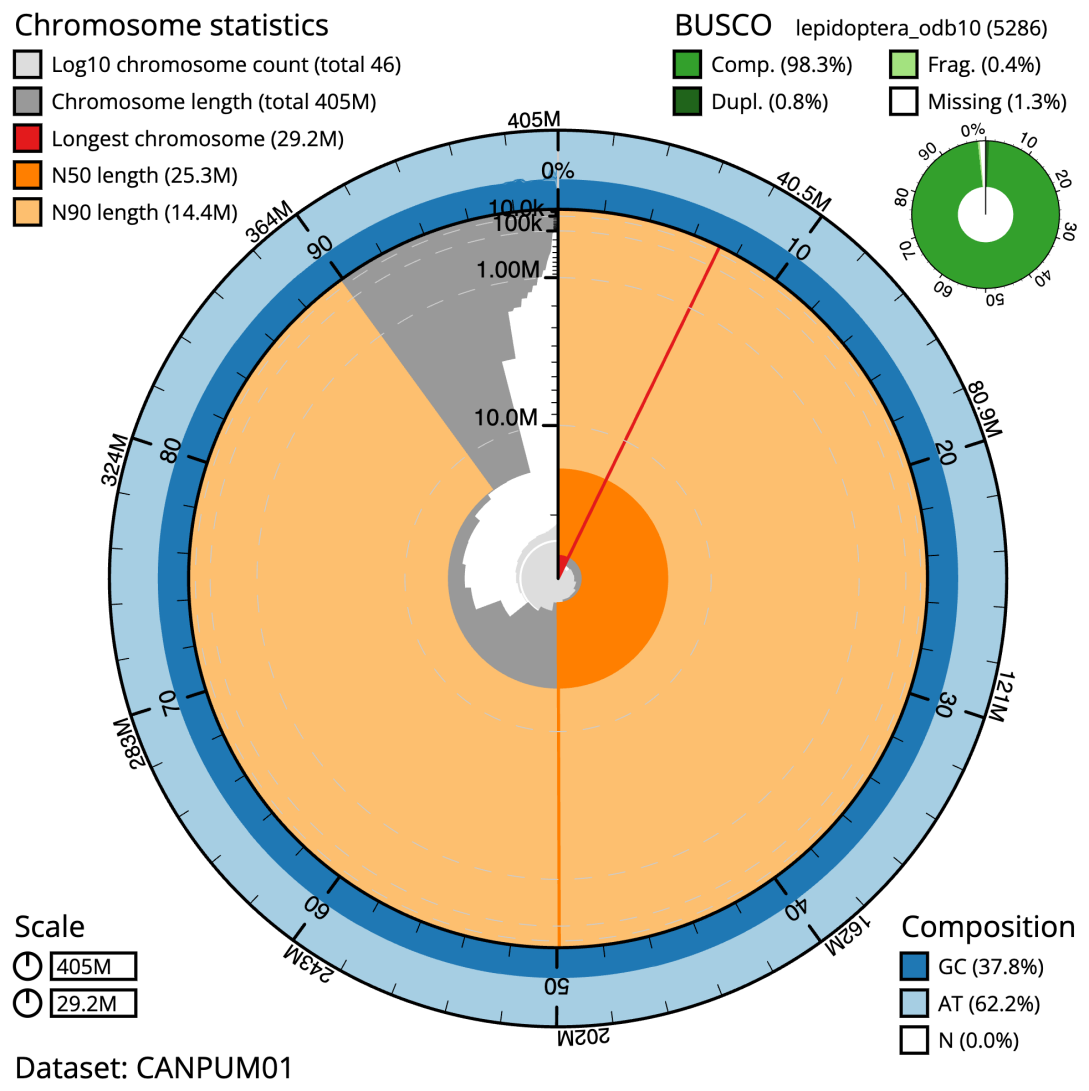
Genome assembly of *Thera obeliscata*, ilTheObel3.1: metrics. The BlobToolKit Snailplot shows N50 metrics and BUSCO gene completeness. The main plot is divided into 1,000 size-ordered bins around the circumference with each bin representing 0.1% of the 404,701,398 bp assembly. The distribution of scaffold lengths is shown in dark grey with the plot radius scaled to the longest scaffold present in the assembly (29,171,840 bp, shown in red). Orange and pale-orange arcs show the N50 and N90 scaffold lengths (25,318,533 and 14,367,994 bp), respectively. The pale grey spiral shows the cumulative scaffold count on a log scale with white scale lines showing successive orders of magnitude. The blue and pale-blue area around the outside of the plot shows the distribution of GC, AT and N percentages in the same bins as the inner plot. A summary of complete, fragmented, duplicated and missing BUSCO genes in the lepidoptera_odb10 set is shown in the top right. An interactive version of this figure is available at https://blobtoolkit.genomehubs.org/view/ilTheObel3.1/dataset/CANPUM01/snail.

The final assembly has a total length of 404.7 Mb in 45 sequence scaffolds with a scaffold N50 of 25.3 Mb ( Table 1). Most (95.8%) of the assembly sequence was assigned to 18 chromosomal-level scaffolds, representing 17 autosomes and the Z sex chromosome. Chromosome-scale scaffolds confirmed by the Hi-C data are named in order of size ( Figure 2– Figure 5; Table 2). The karyotype of *Thera obeliscata* in this sample does not match the expected karyotype of 13 ( Suomalainen, 1965). While not fully phased, the assembly deposited is of one haplotype. Contigs corresponding to the second haplotype have also been deposited. The mitochondrial genome was also assembled and can be found as a contig within the multifasta file of the genome submission.

**Table 1.** Genome data for *Thera obeliscata*, ilTheObel3.1.

Project accession data		
Assembly identifier	ilTheObel3.1	
Species	*Thera obeliscata*	
Specimen	ilTheObel3	
NCBI taxonomy ID	934896	
BioProject	PRJEB56052	
BioSample ID	SAMEA14448147	
Isolate information	ilTheObel3: head and thorax (DNA sequencing) ilTheObel2: head and thorax (Hi-C data)	
Assembly metrics *		*Benchmark*
Consensus quality (QV)	67.1	*≥ 50*
*k*-mer completeness	100%	*≥ 95%*
BUSCO **	C:98.3%[S:97.5%,D:0.8%],F:0.4%,M:1.3%,n:5,286	*C ≥ 95%*
Percentage of assembly mapped to chromosomes	95.8%	*≥ 95%*
Sex chromosomes	Z chromosome	*localised homologous pairs*
Organelles	Mitochondrial genome assembled	*complete single alleles*
Raw data accessions		
PacificBiosciences SEQUEL II	ERR10224924	
Hi-C Illumina	ERR10297815	
Genome assembly		
Assembly accession	GCA_947578465.1	
*Accession of alternate haplotype*	GCA_947581545.1	
Span (Mb)	404.7	
Number of contigs	114	
Contig N50 length (Mb)	7.6	
Number of scaffolds	45	
Scaffold N50 length (Mb)	25.3	
Longest scaffold (Mb)	29.2	

* Assembly metric benchmarks are adapted from column VGP-2020 of “Table 1: Proposed standards and metrics for defining genome assembly quality” from ( Rhie *et al.*, 2021). ** BUSCO scores based on the lepidoptera_odb10 BUSCO set using v5.3.2 C = complete [S = single copy, D = duplicated], F = fragmented, M = missing, n = number of orthologues in comparison. A full set of BUSCO scores is available at https://blobtoolkit.genomehubs.org/view/ilTheObel3.1/dataset/CANPUM01/busco.

**Figure 3.**
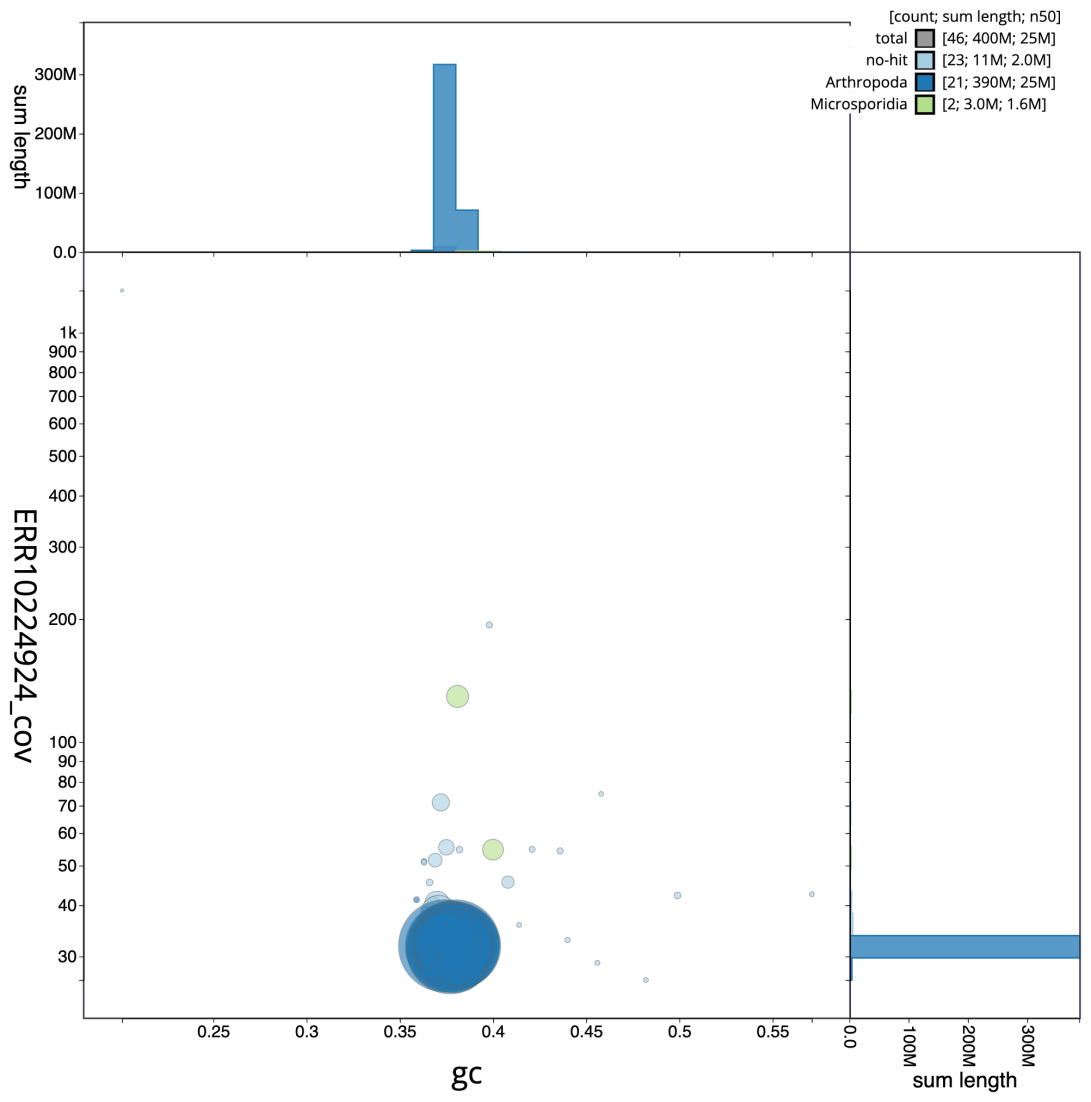
Genome assembly of *Thera obeliscata*, ilTheObel3.1: BlobToolKit GC-coverage plot. Scaffolds are coloured by phylum. Circles are sized in proportion to scaffold length. Histograms show the distribution of scaffold length sum along each axis. An interactive version of this figure is available at https://blobtoolkit.genomehubs.org/view/ilTheObel3.1/dataset/CANPUM01/blob.

**Figure 4.**
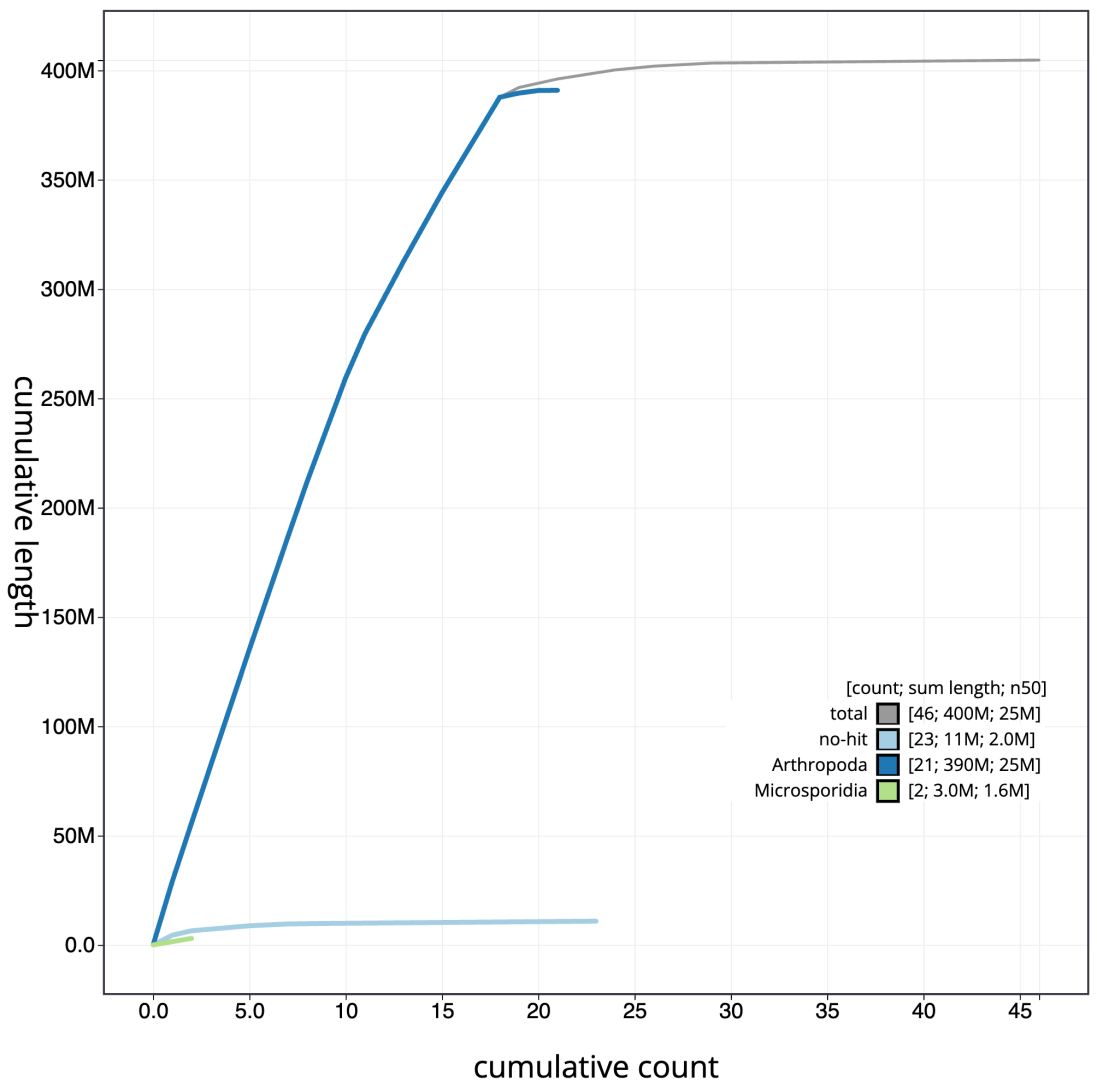
Genome assembly of *Thera obeliscata*, ilTheObel3.1: BlobToolKit cumulative sequence plot. The grey line shows cumulative length for all scaffolds. Coloured lines show cumulative lengths of scaffolds assigned to each phylum using the buscogenes taxrule. An interactive version of this figure is available at https://blobtoolkit.genomehubs.org/view/ilTheObel3.1/dataset/CANPUM01/cumulative.

**Figure 5.**
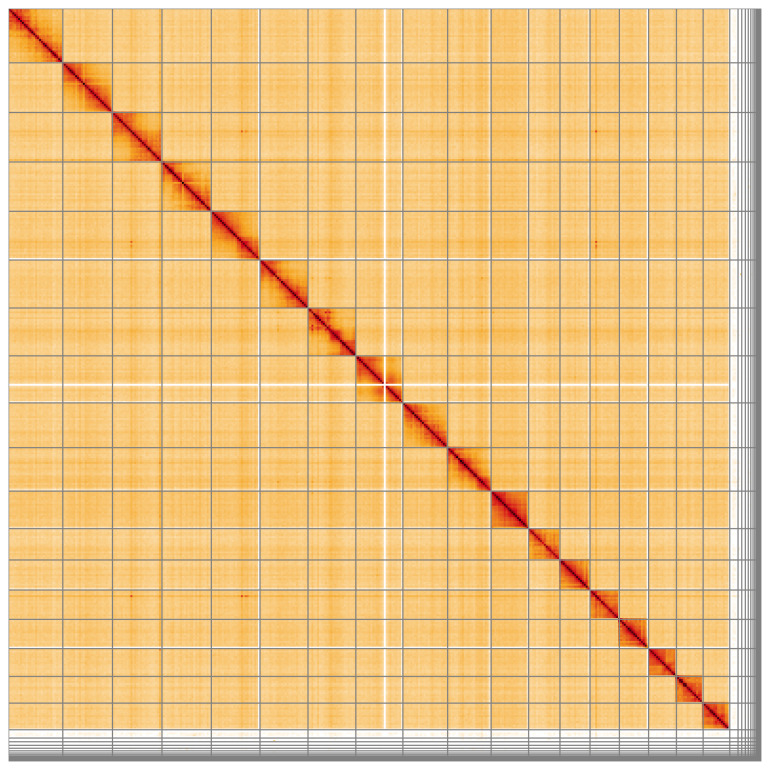
Genome assembly of *Thera obeliscata*, ilTheObel3.1: Hi-C contact map of the ilTheObel3.1 assembly, visualised using HiGlass. Chromosomes are shown in order of size from left to right and top to bottom. An interactive version of this figure may be viewed at https://genome-note-higlass.tol.sanger.ac.uk/l/?d=TUsvOtGYTDKCHidtk4PFTQ.

**Table 2.** Chromosomal pseudomolecules in the genome assembly of *Thera obeliscata*, ilTheObel3.

INSDC accession	Chromosome	Length (Mb)	GC%
OX387912.1	1	29.17	37.5
OX387913.1	2	26.71	38.0
OX387914.1	3	26.59	37.5
OX387915.1	4	26.52	38.0
OX387916.1	5	26.18	38.0
OX387917.1	6	25.79	37.5
OX387918.1	7	25.71	38.0
OX387919.1	8	25.32	37.5
OX387920.1	9	24.07	38.0
OX387921.1	10	23.34	38.0
OX387923.1	11	16.85	38.0
OX387924.1	12	16.17	38.0
OX387925.1	13	15.77	38.0
OX387926.1	14	15.72	38.0
OX387927.1	15	14.95	37.5
OX387928.1	16	14.37	37.5
OX387929.1	17	14.33	37.5
OX387922.1	Z	20.16	38.0
OX387930.1	MT	0.02	20.0

The estimated Quality Value (QV) of the final assembly is 67.1 with *k*-mer completeness of 100%, and the assembly has a BUSCO v5.3.2 completeness of 98.3% (single = 97.5%, duplicated = 0.8%), using the lepidoptera_odb10 reference set ( *n* =5,286).

Metadata for specimens, spectral estimates, sequencing runs, contaminants and pre-curation assembly statistics can be found at https://links.tol.sanger.ac.uk/species/934896.

## Methods

### Sample acquisition and nucleic acid extraction

The *Thera obeliscata* specimens used in this study were collected from Beinn Eighe National Nature Reserve, Scotland, UK (latitude 57.63, longitude –5.35) on 2021-09-09 and 2021-09-10, using a light trap. The specimens were collected and identified by David Lees (Natural History Museum) and were dry frozen (–80°C). One of these specimens (specimen ID NHMUK014543806, individual ilTheObel3) was used for DNA sequencing, and another (specimen ID NHMUK014543809, individual ilTheObel2) was used for Hi-C data.

DNA was extracted at the Tree of Life laboratory, Wellcome Sanger Institute (WSI). The ilTheObel3 sample was weighed and dissected on dry ice. Head and thorax tissue was disrupted using a Nippi Powermasher fitted with a BioMasher pestle. High molecular weight (HMW) DNA was extracted using the Qiagen MagAttract HMW DNA extraction kit. HMW DNA was sheared into an average fragment size of 12–20 kb in a Megaruptor 3 system with speed setting 30. Sheared DNA was purified by solid-phase reversible immobilisation using AMPure PB beads with a 1.8X ratio of beads to sample to remove the shorter fragments and concentrate the DNA sample. The concentration of the sheared and purified DNA was assessed using a Nanodrop spectrophotometer and Qubit Fluorometer and Qubit dsDNA High Sensitivity Assay kit. Fragment size distribution was evaluated by running the sample on the FemtoPulse system.

### Sequencing

Pacific Biosciences HiFi circular consensus DNA sequencing libraries were constructed according to the manufacturers’ instructions. DNA sequencing was performed by the Scientific Operations core at the WSI on a Pacific Biosciences SEQUEL II (HiFi) instrument. Hi-C data were also generated from head and thorax tissue of ilTheObel2 using the Arima2 kit and sequenced on the Illumina NovaSeq 6000 instrument.

### Genome assembly, curation and evaluation

Assembly was carried out with Hifiasm ( Cheng *et al.*, 2021) and haplotypic duplication was identified and removed with purge_dups ( Guan *et al.*, 2020). The assembly was then scaffolded with Hi-C data ( Rao *et al.*, 2014) using YaHS ( Zhou *et al.*, 2023). The assembly was checked for contamination and corrected as described previously ( Howe *et al.*, 2021). Manual curation was performed using HiGlass ( Kerpedjiev *et al.*, 2018) and Pretext ( Harry, 2022). The mitochondrial genome was assembled using MitoHiFi ( Uliano-Silva *et al.*, 2023), which runs MitoFinder ( Allio *et al.*, 2020) or MITOS ( Bernt *et al.*, 2013) and uses these annotations to select the final mitochondrial contig and to ensure the general quality of the sequence.

A Hi-C map for the final assembly was produced using bwa-mem2 ( Vasimuddin *et al.*, 2019) in the Cooler file format ( Abdennur & Mirny, 2020). To assess the assembly metrics, the *k*-mer completeness and QV consensus quality values were calculated in Merqury ( Rhie *et al.*, 2020). This work was done using Nextflow ( Di Tommaso *et al.*, 2017) DSL2 pipelines “sanger-tol/readmapping” ( Surana *et al.*, 2023a) and “sanger-tol/genomenote” ( Surana *et al.*, 2023b). The genome was analysed within the BlobToolKit environment ( Challis *et al.*, 2020) and BUSCO scores ( Manni *et al.*, 2021; Simão *et al.*, 2015) were calculated.

Table 3 contains a list of relevant software tool versions and sources.

**Table 3.** Software tools: versions and sources.

Software tool	Version	Source
BlobToolKit	4.1.5	https://github.com/blobtoolkit/blobtoolkit
BUSCO	5.3.2	https://gitlab.com/ezlab/busco
Hifiasm	0.16.1-r375	https://github.com/chhylp123/hifiasm
HiGlass	1.11.6	https://github.com/higlass/higlass
Merqury	MerquryFK	https://github.com/thegenemyers/MERQURY.FK
MitoHiFi	2	https://github.com/marcelauliano/MitoHiFi
PretextView	0.2	https://github.com/wtsi-hpag/PretextView
purge_dups	1.2.3	https://github.com/dfguan/purge_dups
sanger-tol/genomenote	v1.0	https://github.com/sanger-tol/genomenote
sanger-tol/readmapping	1.1.0	https://github.com/sanger-tol/readmapping/tree/1.1.0
YaHS	yahs-1.1.91eebc2	https://github.com/c-zhou/yahs

### Wellcome Sanger Institute – Legal and Governance

The materials that have contributed to this genome note have been supplied by a Darwin Tree of Life Partner. The submission of materials by a Darwin Tree of Life Partner is subject to the **‘Darwin Tree of Life Project Sampling Code of Practice’**, which can be found in full on the Darwin Tree of Life website here. By agreeing with and signing up to the Sampling Code of Practice, the Darwin Tree of Life Partner agrees they will meet the legal and ethical requirements and standards set out within this document in respect of all samples acquired for, and supplied to, the Darwin Tree of Life Project. 

 Further, the Wellcome Sanger Institute employs a process whereby due diligence is carried out proportionate to the nature of the materials themselves, and the circumstances under which they have been/are to be collected and provided for use. The purpose of this is to address and mitigate any potential legal and/or ethical implications of receipt and use of the materials as part of the research project, and to ensure that in doing so we align with best practice wherever possible. The overarching areas of consideration are: 

 Further, the Wellcome Sanger Institute employs a process whereby due diligence is carried out proportionate to the nature of the materials themselves, and the circumstances under which they have been/are to be collected and provided for use. The purpose of this is to address and mitigate any potential legal and/or ethical implications of receipt and use of the materials as part of the research project, and to ensure that in doing so we align with best practice wherever possible. The overarching areas of consideration are: 

• Ethical review of provenance and sourcing of the material

• Ethical Legality of collection, transfer and use (national and international) 

Each transfer of samples is further undertaken according to a Research Collaboration Agreement or Material Transfer Agreement entered into by the Darwin Tree of Life Partner, Genome Research Limited (operating as the Wellcome Sanger Institute), and in some circumstances other Darwin Tree of Life collaborators.

## Data Availability

European Nucleotide Archive: *Thera obeliscata*. Accession number PRJEB56052; https://identifiers.org/ena.embl/PRJEB56052. ( Wellcome Sanger Institute, 2022) The genome sequence is released openly for reuse. The *Thera obeliscata* genome sequencing initiative is part of the Darwin Tree of Life (DToL) project. All raw sequence data and the assembly have been deposited in INSDC databases. The genome will be annotated using available RNA-Seq data and presented through the Ensembl pipeline at the European Bioinformatics Institute. Raw data and assembly accession identifiers are reported in Table 1.

## Author information

Members of the Natural History Museum Genome Acquisition Lab are listed here: https://doi.org/10.5281/zenodo.4790042.

Members of the Darwin Tree of Life Barcoding collective are listed here: https://doi.org/10.5281/zenodo.4893703.

Members of the Wellcome Sanger Institute Tree of Life programme are listed here: https://doi.org/10.5281/zenodo.4783585.

Members of Wellcome Sanger Institute Scientific Operations: DNA Pipelines collective are listed here: https://doi.org/10.5281/zenodo.4790455.

Members of the Tree of Life Core Informatics collective are listed here: https://doi.org/10.5281/zenodo.5013541.

Members of the Darwin Tree of Life Consortium are listed here: https://doi.org/10.5281/zenodo.4783558.
