# Supplementary material for: The genome sequence of the Grey Pine Carpet, Thera obeliscata (Hübner, 1787)
Source: Wellcome Open Res. 2023 Sep 19;8:408. [Version 1] doi: 10.12688/wellcomeopenres.20005.1 (PMC11099514; doi:10.12688/wellcomeopenres.20005.1)
Supplement: Peer review history

# Reviewer report: Reviewer response for version 1

**DOI:** 10.21956/wellcomeopenres.22151.r76265

**Published:** 2024-05-16

**Authors:** Jerome Hui

**Affiliations:** 1 The Chinese University of Hong Kong, Hong Kong, Hong Kong

Lees and colleagues report the genome sequence of a male grey pine carpet *Thera obeliscata *(Hubner, 1787). This moth species can be commonly found in Britain. Molecular data of this species are mainly confined to COI sequences deposited to the NCBI database, and it is good to begin to see these information are included and written in the background of the report (as well as with certain level of comparison to other genomes generated by the Darwin Tree of Life). This new genome resource will be useful for further studies, and to name some, such as revealing its population structure and relationship with the host plants, understanding the effect of climate change and/or anthropogenic activities on them, as well as depicting their evolutionary relationships with other lepidopterans.

 This genome resource is excellent from the summary statistics, with high BUSCO numbers (98.3%), high sequence continuity (scaffold N50), and majority of sequences contained on the 18 pseudochromosomes (plus mitochondrion). To sum up, this is another valuable contribution by the Darwin Tree of Life.

Are sufficient details of methods and materials provided to allow replication by others?

Yes

Is the rationale for creating the dataset(s) clearly described?

Yes

Are the datasets clearly presented in a useable and accessible format?

Yes

Are the protocols appropriate and is the work technically sound?

Yes

Reviewer Expertise:

Genomics, evolution, invertebrates

I confirm that I have read this submission and believe that I have an appropriate level of expertise to confirm that it is of an acceptable scientific standard.

# Reviewer report: Reviewer response for version 1

**DOI:** 10.21956/wellcomeopenres.22151.r67455

**Published:** 2023-11-06

**Authors:** Paul Frandsen

**Affiliations:** 1 Brigham Young University-Idaho, Rexburg, Idaho, USA

This is a nice contribution of a species that is widespread, but has nevertheless experienced recent declines in population numbers. It also appears that the species boundaries are a bit unclear and a genome may help with those determinations. My only critique is that the discussion on the mitochondrial divergence between the grey carpet and its potential sister species could be made a bit more clear.

Are sufficient details of methods and materials provided to allow replication by others?

Yes

Is the rationale for creating the dataset(s) clearly described?

Yes

Are the datasets clearly presented in a useable and accessible format?

Yes

Are the protocols appropriate and is the work technically sound?

Yes

Reviewer Expertise:

Biodiversity genomics, bioinformatics, systematics

I confirm that I have read this submission and believe that I have an appropriate level of expertise to confirm that it is of an acceptable scientific standard.
